# Tegument protein control of latent herpesvirus establishment and animation

**DOI:** 10.1186/2042-4280-2-3

**Published:** 2011-02-08

**Authors:** Rhiannon R Penkert, Robert F Kalejta

**Affiliations:** 1 Institute for Molecular Virology, McArdle Laboratory for Cancer Research, and Cell and Molecular Biology Training Program, University of Wisconsin-Madison, Madison, WI, 53706, USA

## Abstract

Herpesviruses are successful pathogens that infect most vertebrates as well as at least one invertebrate species. Six of the eight human herpesviruses are widely distributed in the population. Herpesviral infections persist for the life of the infected host due in large part to the ability of these viruses to enter a non-productive, latent state in which viral gene expression is limited and immune detection and clearance is avoided. Periodically, the virus will reactivate and enter the lytic cycle, producing progeny virus that can spread within or to new hosts. Latency has been classically divided into establishment, maintenance, and reactivation phases. Here we focus on demonstrated and postulated molecular mechanisms leading to the establishment of latency for representative members of each human herpesvirus family. Maintenance and reactivation are also briefly discussed. In particular, the roles that tegument proteins may play during latency are highlighted. Finally, we introduce the term animation to describe the initiation of lytic phase gene expression from a latent herpesvirus genome, and discuss why this step should be separated, both molecularly and theoretically, from reactivation.

## Review

### Herpesvirus Lytic and Latent Infections

Herpesviruses are large double-stranded DNA viruses with a unique virion morphology consisting of a genome-containing capsid, a proteinaceous tegument, and a lipid envelope. Human herpesviruses are divided into three families (alpha, beta, and gamma) based on tissue tropism and sequence similarity [1]. The amplification of virus within an infected cell or host is accomplished by productive, lytic infection where, upon entry into a susceptible cell (Table 1), a specific cascade of viral gene expression is activated, the genome is replicated to high levels, and infectious progeny virions are assembled and released. The lytic cascade of herpesvirus gene expression initiates with the synthesis of the immediate early (IE) genes. Early and late gene expression follows [1]. Provocatively, unlike other large DNA viruses, many herpesvirus IE genes are not controlled by promoters that are efficient and constitutively active within the context of the viral genome. Rather, viral transactivator proteins incorporated into the virion tegument and released into the cell upon infection play critical roles in the activation of viral IE gene expression [2-5]. Such a mechanism permits a far greater level of regulation than a simple constitutive promoter would allow.

**Table 1:** Cells that support the different types of human herpesvirus infections.

Family	Virus	Productive (Lytic) Replication	Site of Latency
α	HSV-1	Epithelial and keratinocyte	Neuron
	
	HSV-2	Epithelial and keratinocyte	Neuron
	
	VZV	Epithelial, keratinocyte, T cell, sebocyte, monocyte, endothelial, Langerhans and PBMC	Neuron

β	HCMV	Macrophage, dendritic, endothelial, smooth muscle, epithelial and fibroblast	CD34+ HSC, monocyte
	
	HHV-6	T cell	BMP
	
	HHV-7	T cell	T cell

γ	EBV	B cell and epithelial	B cell
	
	KSHV	Lymphocyte	B cell

Common cell types in which the human herpesviruses, grouped by family, initiate productive lytic infection or establish latency are listed. PBMC, peripheral blood mononuclear cells. HSC, hematopoietic stem cell. BMP, bone marrow progenitor.

The productive, lytic cycle is not the only possible outcome upon viral infection of an individual cell. In certain cell types (Table 1), herpesvirus infections establish the viral genomes in the nucleus but a productive round of replication is not completed in a timely manner. In such cells, a different, significantly smaller subset of viral genes is expressed. Importantly, because these infected cells maintain the potential to undergo productive replication at some later time after receiving the appropriate stimulus, this type of infection is described as latency. The resumption and completion of productive, lytic replication after a period of latency is called a reactivation event [1]. Both the restriction of substantial viral gene expression and the maintenance over time of the viral genome during latency allow herpesviral infections to persist for the life of the host even in the face of intense immune surveillance. Reactivation events allow for dissemination throughout and among hosts. While drugs that suppress lytic replication are available [6,7], treatments for latently infected cells currently do not exist. As controlling or curing a herpesvirus infection would require modulation or elimination of the reservoir of latently infected cells, understanding the molecular mechanisms that govern latency is of utmost importance.

### Latency is Cell Type Specific

Cell types that support latent infection with different herpesviruses are highly exclusive and non-overlapping. The alphaherpesviruses, Herpes Simplex Virus type 1 and 2 (HSV-1 and -2) and Varicella Zoster Virus (VZV) establish latency in neurons [8,9]. The betaherpesviruses Human Cytomegalovirus (HCMV), and Human Herpesvirus -6 and -7 (HHV-6 and -7) establish latency in different subsets of hematopoietic cells; HCMV in hematopoietic stem cells [10-12], HHV-6 in bone marrow progenitor cells [13,14] and HHV-7 in T-cells [14,15]. The gammaherpesviruses Epstein Barr Virus (EBV) and Kaposi's Sarcoma Associated Herpesvirus (KSHV) establish latency in B cells [16-18]. Interestingly, most of these viruses have a broader tropism for lytic replication than latent infections, with many herpesviruses being limited to a single cell type in which latency can be established. The specificity for latent infections seems to indicate that each individual cell type contributes important factors that promote latency. This review presents our interpretations of the molecular mechanisms that control herpesvirus latency. For independent views, readers are directed to several recent literature surveys focusing on HSV-1 [19-23], HCMV [24-28], or EBV [29-33].

The systems used to study alpha-, beta-, and gammaherpesvirus latency vary dramatically. A valuable animal model for HSV-1 latency exists, but *in vitro *models [34-38] receive less attention. Thus the genetics of HSV-1 latency is established (i.e. which open reading frames are required for latency), but the molecular/cellular biology (i.e. how the products of the open reading frames actually promote latency) is less well understood. An *in vitro *model for HCMV latency is in its infancy. Despite its technical difficulties, it is amenable to both genetic and molecular analysis. Many cell lines exist to study the maintenance and reactivation of EBV latency, but the difficulty in making high titer virus stocks makes studying the establishment phase challenging. Animal models for HCMV and EBV latency do not exist, although certain aspects can be studied in immunodeficient humanized mice [39,40]. The general lack of animal models for these viruses makes it difficult to determine the true physiologic relevance of *in vitro *studies.

One clear component of latency for each virus is the silencing of lytic phase gene expression. Interestingly, in the absence of tegument transactivators, most if not all viral IE promoters are poorly activated even in cell types where lytic infection occurs and that presumably have all the required cellular activating transcriptional factors for high-level lytic gene expression [2,41]. Thus, the roles of tegument proteins and cellular transcriptional repressors in herpesvirus latency have recently received increased scrutiny. In the sections below, we review the current knowledge of how viral IE gene expression from incoming viral genomes is initially repressed in latently infected cells, and briefly address the maintenance of that repression. Finally, we examine the initial events that may resuscitate latent viral genomes (an event we term animation), and how they relate to full reactivation and infectious progeny virion production.

### Establishing Latent HSV-1 Infections

Establishment of herpesviral latency can be operationally defined as the delivery of the viral genome to the nucleus without the initiation of a productive infection. HSV-1 establishes latency upon infection of a subset of neurons found in trigeminal ganglia, but productively replicates in other types of neurons found in these structures [42-48]. Productive replication within the trigeminal ganglia increases the number of latently infected neurons, but is not absolutely required for the establishment of latency [49-53]. Debate remains as to whether or not neurons destined to establish a latent infection may initially express lytic phase genes and then subsequently extinguish them. Inferred activity of an ectopic ICP0 promoter driving expression of the Cre recombinase and the subsequent Cre-mediated activation of a LacZ reporter from the cellular genome has been used to argue that no more than one-third of latently-infected neurons may have experienced ICP0 expression sometime during the infection [44]. However, the correct Cre-mediated recombination events were not confirmed in those experiments, and the proper IE regulation of the ectopic ICP0 promoter was not examined. Nevertheless, the ectopic ICP0 promoter apparently was never activated in the majority of latently infected neurons [44], indicated that in most (if not all) cells, the very first class of lytic genes, the IE genes, are not expressed upon neuronal infection where latency is established. To appreciate how HSV-1 IE genes may be silenced during the establishment of latency, we must understand how they are activated upon lytic infection.

HSV-1 IE gene expression is activated by a complex consisting of the viral tegument protein VP16, which is delivered to the cell upon entry, and two cellular proteins, host cell factor 1 (HCF) [54-56] and the POU homeodomain protein Oct-1 [57-59]. Tegument-delivered VP16 encounters HCF in the cytoplasm, and this association is absolutely required for VP16 translocation to the nucleus. Tegument-delivered VP16 remains in the cytoplasm of infected cells if binding to HCF is disrupted by mutation, or if the nuclear localization sequence (NLS) of HCF is deleted [60]. Under these circumstances, viral IE gene expression is inhibited. Once in the nucleus, the VP16/HCF pair interacts with Oct-1 associated with TAATGARAT motifs (where R is a purine) found in HSV-1 IE promoters [61]. Now tethered to viral genomes, the VP16/HCF/Oct-1 complex activates viral gene expression by recruiting cellular RNA Polymerase II and by modulating both histone occupancy and chromatin structure of the viral genome [62].

VP16 contains a prototypical acidic activation domain [63] that interacts with RNA Polymerase II, as well as several cellular components of the basal transcriptional machinery, including transcription factor IIB (TFIIB) and IIH (TFIIH), TATA-binding protein (TBP) and other transcription associated factors (TAFs) [56,64-68]. Complex association with the TAATGARAT sequences orients the preinitiation complex facilitating transcriptional initiation. A specific VP16 mutant allele that lacks the acidic activation domain termed RP5 is unable to recruit the basal transcriptional machinery to viral IE promoters [62]. The RP5 virus exhibits a severe growth defect at low multiplicities of infection and is unable to replicate in immunocompetent mice [69].

In addition to recruiting RNA Polymerase II, the VP16/HCF/Oct-1 complex also controls histone occupancy, positioning, and modification at viral IE promoters [62,70,71]. Viral genomes packaged into virions and delivered to the nuclei of infected cells are devoid of cellular histone proteins [72,73]. However, chromatinization occurs rapidly upon nuclear entry and is maintained throughout infection, although histone association with the viral genome is less prevalent and irregularly spaced compared to the cellular genome [21,22,62,72,74-77]. Histones wrap DNA into nucleosomes. The positioning of nucleosomes, as well as the modification of the resident histones, has profound effects on both transcriptional activation and repression.

The dynamics and importance of histone association with herpesviral genomes are just beginning to be appreciated. However, early work has made it clear that the VP16/HCF/Oct-1 complex modulates cellular histone association with viral IE promoters. The acidic activation domain of VP16 interacts with cellular ATP-dependent chromatin remodeling complexes that include the proteins BRG1 and hBRM, which are the mammalian counterparts of the yeast SWI/SNF complex components [78,79]. This results in decreased histone occupancy at viral IE promoters. The RP5 mutant virus is unable to recruit these chromatin remodeling complexes and displays increased histone association with viral IE promoters [62]. HCF interaction with the histone chaperone Asf1b may also play a role in regulating viral genome chromatinization [71].

In addition to controlling the occupancy and positioning of cellular histones on viral genomes, the VP16/HCF/Oct-1 complex also regulates their post-translational modifications. VP16 interacts with the cellular histone acetyltransferases (HATs) CBP and p300, and increases the acetylation of histones associated with viral IE promoters [78,80-83]. Specifically, markers of active euchromatin such as H3K9/K14 acetylation and H3K4 methylation are induced during lytic infection [62,72,76,77]. Interestingly, neither p300 nor CBP activity appear to be required for HSV-1 IE gene expression [84], indicating that either other cellular HATs can compensate, or that histone occupancy is quantitatively more important for controlling viral IE gene expression than is histone modification. In summary, it is becoming increasingly clear that part of the mechanism through which VP16 activates HSV-1 IE gene expression is through reducing the overall level, ensuring the proper positioning, and facilitating the activation-linked modification of histones that become associated with viral promoters upon entry of the genome into the nucleus. Indeed, chromatinization of the viral genome can be a significant barrier to HSV-1 IE gene expression and the initiation of lytic infection that is overcome by the various functions of VP16.

As mentioned above, viral IE genes are likely not expressed at the start of an HSV-1 latent infection. Presumably, this must occur through either a loss of VP16-mediated enhancement, a dominant block to IE gene expression even in the presence of VP16 function, or a combination of both mechanisms. Analyzing the molecular details of the establishment of HSV-1 latency has been monumentally difficult because of the heavy reliance on animal model systems. Mouse and rabbit model systems provide quantitative readouts for various latency parameters, and are invaluable in the analysis of viral genetics in a holistic view of HSV-1 latency. However, in these systems it is either difficult or impossible to perform a detailed molecular analysis, or to divorce lytic replication from latency competence. Animal models require initial lytic infection at epithelial sites (for example, the eye) to seed ganglia with infectious virus that becomes amplified through more lytic replication prior to or concomitant with establishing latency in a subset of sensory neurons. Reactivations scored in animals after various stresses require viruses competent for productive, lytic infection. Alternatively, reactivation can be monitored in explanted neurons. Unfortunately, the explantation process significantly alters neurons, complicating the interpretation of results.

The literature pertaining to HSV-1 latency is enormous and often contradictory, and a full-scale review of it is not our intent here. Below we attempt to address and review a specific aspect of HSV-1 latency, the initial silencing of viral IE gene expression during the establishment of latency. Evolution has provided HSV-1 a mechanism to regulate viral IE gene expression by the use of promoters that are not constitutively active in their native locales, but require a trans-acting, tegument-delivered viral transcription factor (VP16) for their activation. Here we revisit a simple question that, although previously pondered [4,60,85,86] deserves, in our opinion, increased scrutiny: is this potential regulation point used to modulate establishment of latency?

**Figure 1:**
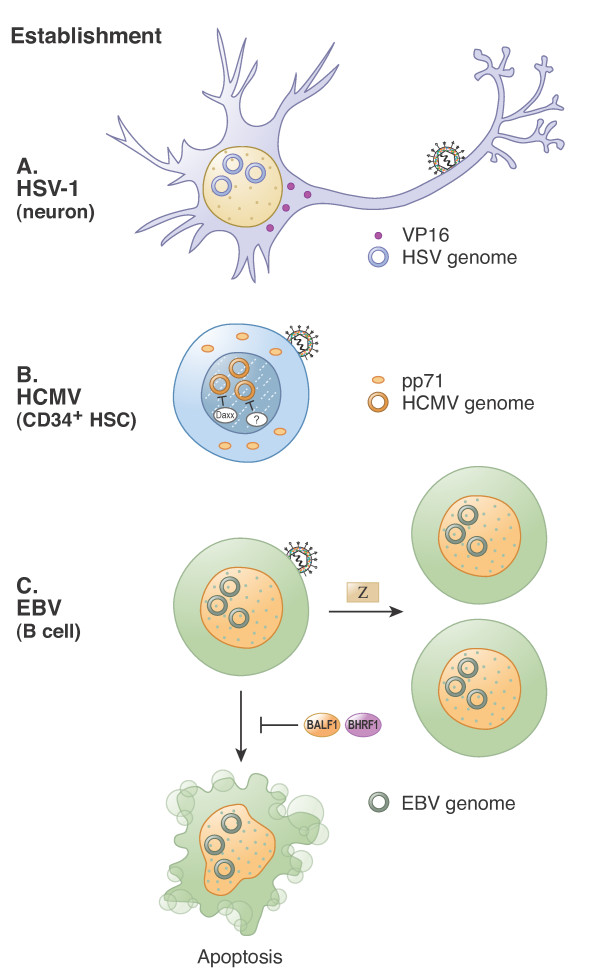
**Establishment of herpesvirus latency**. **A.** Herpes Simplex Virus Type 1 (HSV-1). Infection of a sensory neuron allows for nuclear entry of viral DNA but not the tegument transactivator VP16. Viral immediate early (IE) genes are silenced. **B.** Human Cytomegalovirus (HCMV). Infection of a CD34+ hematopoietic progenitor cell allows for nuclear entry of viral DNA but not the tegument transactivator pp71. Viral IE genes are silenced by Daxx and an unidentified (?) trans-dominant, HDAC-independent mechanism. **C.** Epstein-Barr Virus (EBV). Infection of a memory B cell allows for nuclear entry of viral DNA. Tegument transactivators for EBV are uncharacterized. At least one IE gene (Z) and two early genes (BALF1 and BHRF1) are expressed. Z promotes B cell proliferation and BALF1/BHRF1 inhibit apoptosis, both of which appear to be required for the efficient establishment of latency. Z is unable to fully activate lytic phase gene expression because the viral genome is unmethylated.

To answer this, one must determine if VP16 is or has the potential to be active in sensory neurons at a time when HSV-1 establishes latency. For tegument-delivered VP16 to activate viral gene expression it must pair with HCF [60,87] in the cytoplasm and then translocate to the nucleus. HCF is expressed in neurons. However, unlike other cell types where the protein is mainly nuclear, in neurons, HCF is cytoplasmic [86], perhaps sequestered there through its association with proteins called Zhangfei [88,89] and Luman [90,91], and is often found in association with the Golgi apparatus [92]. This would seem to indicate that nuclear import of VP16 is most likely impaired in neurons. Cytoplasmic sequestration of VP16 may have a direct (and positive) effect on HSV-1 latency as the IE promoters of latent genomes are highly chromatinized, and the associated histones have heterochromatin-like marks, including the absence of acetylation and the presence of repressive modifications, such as H3K9 tri-methylation [93-95]. Such a configuration would favor the transcriptional repression observed and likely be required for the establishment of latency. In addition to the loss of VP16 function, low levels of Oct-1 [96] or differing ratios of Oct protein family members [97] may also decrease the likelihood of IE gene expression in neurons. Thus, it appears that VP16 function could be compromised during the establishment of latency (Figure 1A). Whether this alone, or other mechanisms in addition to or instead of impaired VP16 function, contribute to the absence of viral IE gene expression observed when HSV-1 establishes latency remains to be demonstrated experimentally.

### Maintaining and Reactivating Latent HSV-1 Infections

Maintaining a latent infection requires that the viral genome be perpetuated and the cell kept alive. As neurons are non-dividing, genome replication and faithful partitioning to daughter cells upon division do not represent significant issues for HSV-1 latency. Perhaps to avoid cell death by apoptotic or immune measures, HSV-1 severely restricts viral gene expression during latency. The only major transcript detected is called the LAT for latency-associated transcript [98]. The LAT promoter has binding sites for neuronal-specific transcription factors [99,100] and, unlike viral lytic phase promoters, is associated with histones exhibiting marks of active euchromatin during latency [101]. Although there are open reading frames encoded by LAT with the potential to encode proteins, such proteins have never been reproducibly detected [102]. Rather, LAT is thought to be processed into microRNAs (miRNAs), at least one of which has the potential to translationally silence mRNAs that encode the viral IE gene ICP0 [103-108]. Like other viral IE proteins, ICP0 can activate the expression of other viral genes and thus promote the lytic replication cycle [109-112]. ICP0 is found in virions but it is unclear if tegument-delivered ICP0 can activate gene expression, whereas *de novo *expressed ICP0 clearly does. Thus, preventing the synthesis of viral IE proteins appears to be a critical part of maintaining the latent HSV-1 genome.

The presence of LAT can also affect the chromatin structure of viral IE promoters. In the absence of LAT, the normally heterochromatic structure of the IE promoters during latency takes on features more reminiscent of active, euchromatin [94]. It is currently unknown if this is a direct effect of the LAT transcript similar to the way non-coding RNAs can affect chromatic structure [113], or an indirect effect of the ability of LAT to suppress the expression of ICP0, which itself can promote euchromatin structures on viral genomes [114-116]. LAT has also been proposed to have an anti-apoptotic effect [117], but the mechanism through which this might be achieved is unknown. Interestingly, while LAT-null viruses have latency defects, they still establish, maintain, and reactivate latency to a substantial degree [47,118,119]. This may indicate the presence of one or more non-LAT measures of latency maintenance. Indeed, a miRNA not encoded by LAT that has the potential to translationally silence the mRNA for the viral IE protein ICP4 has also been detected in latently infected cells [103]. Thus it would appear that a major strategy to maintain HSV-1 latency is simply to curtail inappropriate reactivation events by preventing the translation of any spurious IE messages that might be generated. However, direct repression of viral lytic gene expression and/or inhibition of apoptosis may also occur.

**Figure 2:**
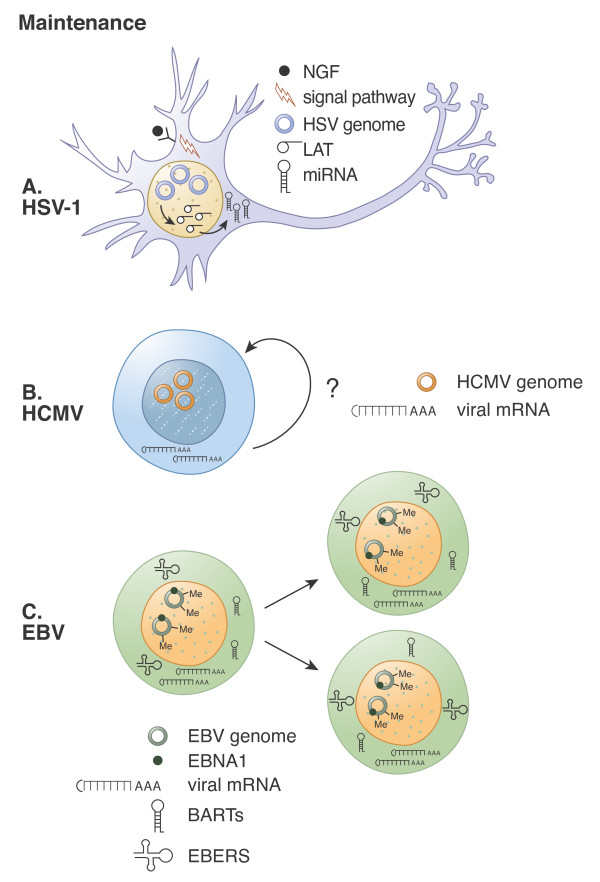
**Maintenance of herpesvirus latency**. **A.** HSV-1. Viral latency associated transcripts (LAT) encode micro RNAs (miRNA) that suppress the expression of viral IE proteins. Nerve growth factor (NGF) induced signaling also helps maintain latency *in vitro*. Non-dividing neurons do not require a mechanism to replicate or faithfully partition viral genomes. **B.** HCMV. The contributions of viral mRNAs/transcripts detected during latency (CTLs, LUNA, UL138, US28, vIL10) to the establishment, maintenance, animation, or reactivation from latency have not been fully characterized. Whether or not latently infected progenitor cells divide or self-renew (arrow with question mark) is not known, thus the need for (or presence of) replication or partition functions is also unclear. **C.** EBV. The viral EBNA1 protein provides replication and partition functions required to maintain latency in dividing B cells. Different types of EBV latency also express other latent genes whose functions appear to be proliferation induction, apoptosis inhibition and immune evasion. EBER and BART transcripts are also expressed during latency. EBERs inhibit protein kinase R (PKR) to maintain translational proficiency, and BARTs are processed into miRNAs. Viral genome methylation prohibits lytic phase gene expression.

Other control measures in addition to LAT (Figure 2A) also help maintain HSV-1 in a latent state. Recent experiments with an *in vitro *model system of primary rat superior cervical ganglia neurons indicate that a signaling cascade starting with nerve growth factor and proceeding through PI3K and Akt is essential to prevent lytic reactivation events [34]. The molecular mechanism through which this pathway suppresses reactivation has not been described. However, the *in vitro *model system utilized appears much more amenable to molecular studies than existing animal models, so the prospects for a more detailed dissection of how this pathway maintains the viral genome in a latent state are appealing.

Reactivation mechanisms of latent HSV-1 infections have been notoriously controversial. Even the stage of infection at which the reactivation event initiates (IE gene expression or viral DNA replication) has been debated [120-124]. Because the latent genome is without an accompanying tegument, it has always been assumed that reactivation of a lytic infection from latency must be fundamentally different from the *de novo *initiation of the lytic replication program upon infection of a susceptible cell type. Complex animal experiments with complicated interpretations have been used to explore HSV-1 reactivation. Most or all of these depend on the ability of mutant viruses to reactivate from latency and complete productive replication, often in explant cultures from HSV-1 infected animals.

ICP0 is the protein most often described as an inducer of reactivation. ICP0 mutant viruses clearly fail to reactivate from animal models of HSV-1 latency [125,126]. However, they also have severe defects during *de novo *lytic infections [127,128]. Thus it is unclear if the latency phenotype of ICP0 mutants is due to defects in the initiation of lytic phase gene expression from a latent viral genome, or from the failure to complete the lytic replication cycle after it has been efficiently started (or both). Furthermore, differing readouts of reactivation (explant vs. *in vivo*) give different results as to the requirement for ICP0 during reactivation [125,126,129]. Recently, VP16 has been proposed to be an inducer of reactivation [130], even though earlier work indicated that a VP16 mutant with a non-functional acidic activation domain (in1814) can establish, maintain, and reactivate from latency [131,132] despite being impaired in its ability to initiate a *de novo *lytic infection [41]. Readers are directed to numerous recent reviews with a more detailed and complete examination of mechanisms controlling HSV-1 latency maintenance and reactivation [19,21-23].

### Animating Latent HSV-1 Infections

Two significant obstacles hinder our understanding of HSV-1 latency. The first is the underutilization of suitable *in vitro *systems [34-38] relative to the heavy reliance upon animal models, valuable as they are, for all levels of latency experimentation. The recent resurrection of a tractable cell culture model for HSV-1 latency [34] should catalyze additional molecular studies. The second is the marriage of the initial events that awaken latent genomes with the completion of productive viral replication. This is necessitated by the term reactivation, which requires productive replication as an endpoint, and therefore mixes both inciting and propagating events under the same moniker. Based on the concept that initiating reactivation and completing reactivation represent two separate events [129,133], we propose the word animation to describe the very first lytic phase event that occurs from latent viral genomes. This theoretical separation has practical application, as it allows one to divorce the completion of productive lytic replication in a previously latent cell from its initiation.

The verb animate means to give life or motion to, thus we define herpesvirus animation as execution of the event that ends latency and initiates the productive replication cycle via reactivation. Unlike designations such as initiation or reactivation that refer to productive infections, animation does not require the eventual generation of infectious virus particles. Applied to latency, animation as a term provides a clear and defined separation between the commencement and completion of a reactivation event. This nomenclature provides concise specific terminology to an important, circumscribed event currently masked by its inclusion with the many subsequent occurrences of reactivation. It allows us to ask what is the animating event in HSV-1 reactivation, and what controls it? Treatment of latently infected mice with acyclovir (an inhibitor of viral DNA replication) concomitant with a reactivation stimulus (heat stress) inhibited late gene expression and reactivation, but did not prevent IE protein production [134]. Thus while viral DNA replication is required for reactivation, it is apparently not required for animation. In similar experiments it was demonstrated that ICP0 mutant viruses could produce IE proteins after heat stress, but did not productively replicate [129]. Thus, while ICP0 is clearly required for reactivation, it apparently is not required for animation.

**Figure 3:**
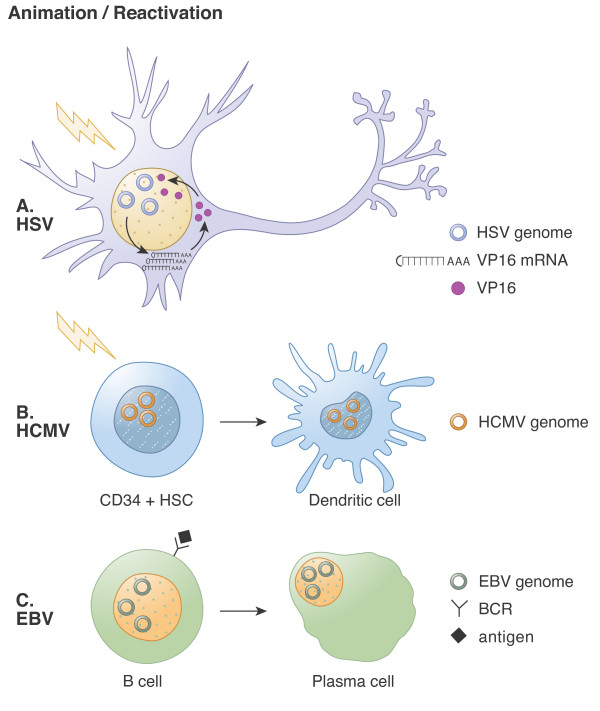
**Animation and reactivation of herpesvirus latency**. **A.** HSV-1. Stress signals (lightning bolt) induce the *de novo *expression of VP16 and its recruitment into the nucleus (likely via HCF) to activate viral IE gene expression. A productive reactivation event follows. **B.** HCMV. Signals (lightning bolt) induce CD34+ cell differentiation into a dendritic cell, inducing animation and subsequent reactivation through unknown molecular mechanisms. **C.** EBV. Activation by antigen stimulation of the B cell receptor (BCR) induces B cell differentiation into a plasma cell, inducing animation and subsequent reactivation through unknown molecular mechanisms.

If the two main protagonists in the reactivation debate (ICP0 and DNA replication) are not responsible for genome animation, than what is? Could VP16, which activates the genome upon *de novo *lytic infection, also be responsible for animating latent HSV-1 genomes? A caveat to this hypothesis is that a specific VP16 mutant virus (in1814) with a 12 bp insertion in the acidic activation domain can efficiently reactivate from latency in explant assays [131,132]. However, this same mutant fails to reactivate in heat stressed mice [130]. As the *in vivo *assay would presumably be more physiologically relevant, it appears that VP16 is required for reactivation, and thus may be required for animation. In a direct test of this model it was observed that viral IE proteins were expressed after heat stress in animals latently infected with an ICP0 mutant virus [129], but not with VP16 mutant viruses [130]. In addition, two different reporter viruses detected VP16 promoter activity after the reactivation stimulus, but prior to IE protein production [130]. While VP16 is expressed as a late gene during *de novo *lytic infection, it apparently is expressed prior to the classically defined viral IE genes in heat stressed mice. Thus, the *de novo *expression of the VP16 protein appears to be the animating event that initiates the reactivation of a productive infection from latency (Figure 3A). It is unclear what changes the stressed cell may undergo that facilitate VP16 expression during latency animation. However, at least some of those changes appear to specifically modulate the activity of the VP16 promoter, as the late promoter that drives expression of the gene encoding the major capsid protein (UL19; VP5) could substitute for the VP16 promoter during lytic infection *in vitro *and during acute ocular infections of mice, but was severely impaired for replication in mouse trigeminal ganglia [130]. Interestingly, this implies that a significant amount of viral replication in the ganglia occurs not by *de novo *lytic infection but by reactivation of (short duration) latent infections, even during the acute phase. In addition, it is likely that cellular stresses also lead to HCF subcellular relocalization to allow the newly expressed VP16 to enter the nucleus and activate viral IE gene expression. Therefore, animation of the HSV-1 genome may be nearly identical for *de novo *lytic infections and for latency reactivations, with the only difference being the source of VP16 (tegument delivered or newly synthesized).

### Establishing Latent HCMV Infections

Numerous viral genes have been reported to be expressed upon *in vitro *infection of CD34+ hematopoietic progenitor cells with HCMV [135-139]. This heterogeneous population of cells represents the most widely accepted and utilized model for experimental HCMV latency. It is clear that the viral IE genes are not among the latently expressed transcripts. Thus it appears that silencing viral IE gene expression occurs as HCMV establishes latency. To appreciate how this occurs, we must understand how IE genes are activated during lytic infection.

Expression of the main viral IE proteins, IE1 and IE2, is controlled by the Major Immediate Early Promoter, or MIEP. Often referred to as "the CMV promoter", the MIEP is constitutively active when found in heterologous constructs such as plasmids, but is surprisingly dependent on viral tegument transactivators for activation in the context of the viral genome [2,140-143]. The major tegument transactivator is pp71 [144]. Upon infection of a cell type permissive for lytic replication, pp71 travels to the nucleus and activates the MIEP by inactivating a cellular intrinsic immune defense that would otherwise silence HCMV gene expression [145]. This defense is mediated by cellular transcriptional co-repressors that localize to promyelocytic leukemia nuclear bodies (PML-NBs) [146-153]. Recombinant viruses with pp71-null or Daxx-binding mutations have severe growth defects and fail to effectively initiate IE gene expression [2,154]. The hallmark event of this cell defense neutralization is the degradation of Daxx [145]. The mechanism through which pp71 induces Daxx degradation is not understood, although proteasome activity and the ability of pp71 to interact with Daxx is required. Interestingly, all evidence points to an ubiquitin-independent route to the proteasome [155,156].

The Daxx-mediated defense silences viral IE gene expression by fostering a transcriptionally repressive chromatin structure on the HCMV genome [157]. Virion-packaged DNA lacks histones [158] but is rapidly chromatinized upon entry into the nucleus [159,160]. Prior to, or in the absence of pp71 function, the histones that become associated with the HCMV genome bear transcriptionally repressive post-translational modifications, such as the absence of acetylation and the presence of H3K9 dimethylation [161-164]. Association with the repressive HP-1 protein is also observed. Knockdown of Daxx by siRNA reduces heterochromatic markings associated with the HCMV genome [157]. When pp71 is present and active, or when Daxx levels are decreased by RNA interference approaches, histones associated with the viral genome are acetylated, a mark of transcriptionally active euchromatin [161-166]. As Daxx associates with histone deacetylases (HDACs), the widely accepted model is that the pp71-mediated degradation of Daxx prevents HDAC association with the HCMV genome and thus facilitates euchromatin formation and transcriptional activity. pp71 also displaces ATRX from Daxx [167], thus further activating IE gene expression. ATRX has homology to the SWI/SNF family of chromatin remodeling proteins [168,169], but it is currently unclear if this protein alters the occupancy or placement of histones at the MIEP.

As mentioned above, IE genes are not expressed at the start of HCMV latency. Presumably, this must occur through a loss of pp71-mediated de-repression, a dominant block to IE gene expression, or a combination of both mechanisms. In the CD34+ cell populations, it appears both mechanisms are used (Figure 1B). pp71 is prevented from degrading Daxx and de-repressing viral gene expression because it fails to localize to the nucleus in infected CD34+ cells [153]. Knockdown of Daxx in these cells, or treatment with the HDAC inhibitor valproic acid rescues IE gene expression upon infection with the AD169 laboratory-adapted strain of HCMV [153]. Thus, the Daxx-mediated intrinsic defense contributes to the silencing of the MIEP that occurs when latency is established. Essentially identical results [152] were obtained in two other cell culture models for quiescent HCMV infections that appear to faithfully mimic most aspects of latency. Differentiation fails to induce reactivation from these quiescently infected cells, though more recent reports suggest treatment with vasoactive intestinal peptide (VIP), an immunomodulatory neuropeptide [170], or phorbol 12-myristate 13-acetate [171], can induce low levels of reactivation.

As the same intrinsic defense that represses IE1 expression during lytic infection prior to pp71 function also silences expression during latency [145,153], it is not surprising that indistinguishable chromatin is assembled on this transcriptionally inert viral locus under these two different conditions. Latent genomes either from *in vitro *infected CD34+ cells or from natural latent infections *in vivo *display unacetylated and H3K9 dimethylated histones and are associated with the HP-1 transcriptional repressor [162,163], similar to what is found during lytic infection in the absence of pp71.

Thus, analogous to HSV-1, sequestration of the virion tegument transactivator protein in the cytoplasm appears to be at least one way that viral genes are silenced at the start of latency. While it is likely that the cytoplasmic localization of HCF prevents VP16 nuclear localization in HSV-1 infected neurons, it is unclear what restricts pp71 nuclear entry during HCMV infection of CD34+ cells. Interestingly, *de novo *expressed pp71 in CD34+ cells localizes to the nucleus [153], so it does not appear that pp71 trafficking is controlled in the same manner as VP16. pp71 cytoplasmic localization is more likely due to a defect in tegument disassembly than to a specific effect on pp71 trafficking because at least one other tegument protein, pp65, is also sequestered in the cytoplasm in undifferentiated NT2 cells quiescently infected with HCMV [172]. pp65 localization upon latent infection of CD34+ cells has not been analyzed. A mechanism for this hypothesized defect in tegument disassembly has not been offered, although it appears that this is not a dominant block imposed by undifferentiated cells, but a recessive trait. Heterologous fusions of undifferentiated and differentiated cells permit tegument-delivered pp71 nuclear localization, leading to the conclusion that differentiated cells possess a dominantly acting factor that drives tegument-delivered pp71 nuclear localization [172]. This fits well with the observed sub-cellular localization of pp71 and IE gene expression competency for matched pairs of undifferentiated and differentiated cells [152].

Interestingly, pp71 cytoplasmic sequestration is not the only mechanism to restrict IE gene expression during establishment of HCMV latency in CD34+ cells. While IE gene expression from the AD169 genome could be rescued by HDAC inhibition, this was not the case during infection with the FIX or TB40/E clinical strains of HCMV [153]. Clinical strains of HCMV have undergone significantly fewer passages *in vitro *and retain a large section of the viral genome (called the ULb' region) that is absent in laboratory-adapted strains such as AD169 [173-175]. In mixed infections between AD169 and a clinical strain, the clinical-strain imposed restriction of viral IE gene expression in the presence of HDAC inhibition was dominant [153]. Whether this clinical strain encoded, trans-dominant HDAC independent restriction of IE gene expression occurs in a cell autonomous or non-cell autonomous manner, and the viral gene responsible for this restriction, is unknown. Also, whether or not this clinical strain function is newly expressed upon viral infection or is a component of the infecting virion has not been determined. The fact that viral DNA damaged by ultraviolet (UV) light, which is unable to support transcription, fails to be maintained in latently infected cultures over time has been used to conclude that viral gene expression is required for the establishment of HCMV latency [136]. While this may be true, viral DNA clearance under these experimental conditions may (also or instead) be the result of the UV-induced damage itself, and not the lack of gene expression. Thus it is currently unclear if the establishment of HCMV latency requires viral latent gene expression or simply the silencing of viral lytic gene expression. Either way, HCMV uses at least two mechanisms to silence viral IE gene expression during the establishment of latency, pp71 cytoplasmic sequestration and an unidentified HDAC-independent block [153].

### Maintaining and Reactivating HCMV Latent Infections

How HCMV maintains latency is poorly understood (Figure 2B). Unlike other betaherpesviruses that have shown the ability to insert their genomes into cellular chromosomes [176-178], the available evidence indicates that latent HCMV genomes are episomal, circular molecules [179]. It is also unclear whether HCMV genomes are replicated during latency. The half-life and dividing potential, *in vivo*, of latently infected CD34+ cells is not known. If latently infected cells fail to divide but have long half-lives, this would mimic the situation with HSV-1 latent infection of neurons. If cells latently infected with HCMV do divide, it would more resemble the situation with EBV, and would require replication and likely partitioning functions for the viral genome during latency. Alternatively, the latent reservoir may be a short-lived non-dividing cell, which would require that HCMV continually re-seed the latent reservoir to achieve life-long persistence. Clearly, these are important questions that to date have not been sufficiently addressed.

While over 80 genes are reported to be expressed in *in vitro *latently infected cells [136,139], only a few are reportedly expressed during natural latent infections *in vivo*. These are the CLTs, US28, vIL10, LUNA, and UL138 loci. Contributions that these loci may (or may not) make to HCMV latency has been recently reviewed [26], so they will only be briefly mentioned here. The CTLs (CMV latency transcripts) represent sense and antisense RNAs from the MIE locus [180,181]. While there is potential for CTLs to act in an antisense or interfering way with viral IE gene expression, no such activity has been demonstrated. Antibodies to proteins hypothetically encoded by the CTLs have been detected in HCMV seropositive patients [182], implying that they might be translated. However, deleting the prominent open reading frame of these transcripts (UL94) did not impair *in vitro *latency [183]. US28 is a chemokine receptor [184,185] whose role during latency has not been studied. vIL10 is a cytokine that may protect latently infected cells against host immune surveillance [137,186,187]. A transcript antisense to the UL81-82 region of the genome encodes an open reading frame termed LUNA for latency unidentified nuclear antigen [138,188]. While LUNA may indeed be a functional protein, this transcript has the potential to modulate *de novo *pp71 expression in an antisense or interfering manner (pp71 is the product of the UL82 gene). Analyses examining the requirement or role for LUNA during latency have not been reported. UL138 encodes a protein that localizes to the Golgi apparatus during lytic infection and is required for the maintenance of latency in some [189], but not other, *in vitro *model systems [187]. A mechanism for how UL138 may regulate latency has not been proposed. A specific HCMV-encoded microRNA, miR-UL112-1, has been proposed as a potential way that viral IE gene expression may be downregulated during latency [190,191], although the expression of this or any other HCMV microRNA during latency has not been reported.

Reactivation of latent HCMV genomes is known to depend upon cell differentiation [162,192-195], although the detailed molecular mechanisms behind this event are not understood. What is known is that latent viral genomes lose their heterochromatic marks and obtain marks of active euchromatin upon the differentiation of CD34+ cells into dendritic cells [162,163]. This seems to indicate that the cellular intrinsic defense that helps establish latency is inactivated during the process of reactivation.

### Animating Latent HCMV Infections

A significant obstacle to our understanding of HCMV latency is the technical difficulties inherent in using CD34+ cells as an *in vitro *model. These cells are heterologous in nature, are difficult or impossible to maintain in an undifferentiated state for even short periods of time, and infect inefficiently, even with clinical strain viruses. Models for how HCMV may animate from latency have included the *de novo *expression of cellular transcription factors specific for the MIEP upon differentiation, or the *de novo *expression of pp71. Both models have their deficiencies. The transactivator model cannot explain how the Daxx-mediated intrinsic defense would be overcome. In this sense it is analogous to a pp71-null virus infection of a differentiated fibroblast, where MIEP activating transcription factors are present but viral IE gene expression is still poor [2]. The *de novo *expression of pp71 prior to IE gene expression upon the differentiation of latently infected CD34+ cells was examined but not detected [188], indicating either that it does not occur, or was below the limit of detection of the assay used. How differentiation triggers HCMV genome animation is still unclear (Figure 3B).

### Establishing Latent EBV Infections

Studies of EBV replication are significantly different from those of HSV-1 and HCMV. Latent EBV infection of B cells transforms them [196] and this represents a cancer burden in the human population [17,30]. Thus, most work on EBV concentrates on the mechanisms through which EBV transforms cells. Consequently, molecular mechanisms that control the initiation of a lytic infection upon primary infection of a cell permissive for productive infection, or the establishment of latency upon the primary infection of a B cell, are poorly understood. For HSV-1 and HCMV, it is clear that infection of cell types fully permissive for productive infection substantially amplifies the amount of virus present. This amplification by *de novo *lytic infection may be important for the establishment of latency because of the accessibility of cell types that support latency. Though a similar process most likely occurs during EBV infection [197], it is currently unclear if such an initial amplification by lytic infection in fully permissive cell types such as epithelial cells is required for the efficient establishment of EBV latency [198]. It is possible that the initial infectious event is a latent infection of a circulating B cell, and that reactivation of that infection could be the source of amplified virus. Thus, it is unclear how much insight into the pathology of the virus is provided by the study of initial events occurring during primary infection of epithelial cells. Such experiments are challenging to perform because infectious stocks of EBV virions are difficult to make and transfer of virus to epithelial cells, either by free virions or by B cell associated virions is inefficient [199-201]. Furthermore, the differentiation state of the epithelial cell appears to impact the outcome of an acute infection [201]. Recent work has determined that an abortive infection of primary epithelial cells resulted in gene expression patterns that were different from infection of epithelial cell lines and primary B cells [200]. However, the gene expression pattern of a *de novo *initiated, productive lytic infection has not been determined, and thus the molecular mechanisms that would account for this gene expression pattern are unknown. Critically, it is not known which or even if tegument proteins activate viral gene expression under these circumstances.

Significantly more is known about the establishment of latency upon primary infection of a B cell (Figure 1C), although the molecular details have generally not been deciphered. Viral IE gene expression is not silenced upon infection of cells destined for latency. At least three lytic phase genes are expressed upon latent infection of primary B cells and are apparently required in order to efficiently establish latency. These are the Bcl-2 homologs BALF1 and BHRF1 [202] and the AP-1 homolog BZLF1 [203,204]. BHRF1 is also expressed during some types of latency [205]. Other reports of more substantial lytic gene expression during the establishment of latency likely represent productive replication in a subset of fully permissive cells found in the peripheral blood mononuclear cells (PBMCs) used for those experiments [206]. Cellular AP-1 is a DNA binding transcription factor, and so is the Z protein (also called Zta, ZEBRA and EB1) encoded by BZLF1 [207]. Z expression reportedly drives the proliferation of quiescent naïve and memory B cells upon infection, and this significantly increases the efficiency with which EBV establishes latency [203]. However, Z-null viruses can still establish latent infections *in vitro *[39,208], so this step appears not to be absolutely required. The unscheduled proliferation induced by Z may be pro-apoptotic, and this may necessitate the expression of BALF1 and BHRF1 proteins. Like their cellular counterpart Bcl-2 [209], these proteins have anti-apoptotic effects [210,211]. Deletion of both BALF1 and BHRF1 inhibited the ability of EBV to latently infect and induce the transformation of primary B cells [203]. The BHRF1 locus also encodes four miRNAs that may enhance the establishment of latency by promoting cellular proliferation and survival [212,213].

The role of Z during the establishment of latency is clearly different from its role during reactivation, when it drives viral early and late gene expression and activates the lytic origin of DNA replication, leading to infectious virion production [29,214]. Early and late lytic phase genes are not expressed during the establishment of latency even though Z is [203,204]. The differential effects of Z on viral gene expression during the establishment and reactivation phases of latency can be explained by the methylation status of the viral genome [215-217]. In the virion, and thus initially upon *de novo *infection, the viral genome is unmethylated. After latency is established, the EBV genome becomes extensively methylated [203]. Though DNA methylation is typically a cellular mark of transcriptional inactivity, EBV has evolved a clever way to overcome methylation-mediated silencing by the cell. The Z protein is able to bind to methylated DNA more strongly than unmethylated DNA [216,217], and Z activates transcription from methylated promoters significantly more than from unmethylated promoters [203,215]. Thus, while Z is expressed during the establishment of latency, the viral genome is unmethylated and so Z cannot activate the expression of early and late viral genes, and therefore under these circumstances, does not induce the lytic phase. Interestingly, another EBV IE gene product, the R protein encoded by the BRLF1 gene, is a transcription factor that preferentially activates unmethylated promoters [216]. There is speculation that R may be more important than Z in initiating *de novo *lytic infection (Shannon Kenney, personal communication). Thus EBV may encode two unique IE proteins with independent activities, one to promote *de novo *lytic infection (R), and one (Z) to promote the establishment and reactivation (see below) of latency.

It is unclear if the expression of Z, BALF1 and BHRF1 at the start of latency requires a tegument protein in a similar manner to the lytic phase IE genes of HSV-1 and HCMV. Transfected HSV-1 or HCMV DNA is capable of initiating a lytic infection, but co-transfection with an expression plasmid for VP16 or pp71 (respectively) increases the efficiency of this process by at least 10-fold, mimicking the effects of the tegument-delivered protein upon *de novo *infection [218,219]. Interestingly, a virus deficient in the EBV tegument protein BNRF1 can enter cells but fails to efficiently establish latency [220]. BNRF1 has recently been shown to bind to Daxx, disrupt its association with ATRX, and stimulate viral gene expression from a co-transfected EBV bacterial artificial chromosome (BAC) construct (Paul Lieberman, personal communication). Thus, EBV tegument proteins may promote the expression of genes required for the establishment of viral latency in a homologous fashion to the manner in which HCMV pp71 promotes the expression of viral genes that initiate lytic infection.

### Maintaining and Reactivating Latent EBV Infections

Latently infected B cells can be generated *in vitro *or isolated from infected patients. EBV transforms and immortalizes latently infected B cells, creating lymphoblastoid cell lines, or LCLs [17,30,196]. These cells divide, and thus EBV must ensure both the replication and the faithful partitioning of its genome during the maintenance of the latent state [221-225]. An enormous amount of literature exists concerning how EBV transforms B cells and maintains latency. As this has been extensively reviewed in several recent manuscripts [29-31,196,226-228], we only briefly describe the general mechanistic concepts here.

In latently infected B cells (Figure 2C) up to nine virally encoded proteins are expressed, these include the EBV nuclear antigens (EBNA-1, -2, -3A, -3B, -3C and -LP) and the latent membrane proteins (LMP-1, -2A and -2B). Different types of latency (e.g. type I, type II, or type III) display different sets of virally expressed genes [32,227,229,230]. EBNA-1 is expressed in all types of latency and plays a central role in maintenance of the viral genome as it is responsible for initiation of episomal DNA replication and segregation during cell division [221,229,231,232]. The other viral proteins contribute to the transformation and immortalization of the infected B cells [226,229,230]. In addition to these viral proteins, non-coding viral RNAs are detected in all latently infected cells, including the EBERs (EBV-encoded RNAs) and the BARTs (BamHI-A rightward transcripts). EBERs inhibit PKR-mediated apoptosis and induce expression of the cellular chemokines IL-6 and IL-10, which promote B cell growth [213,233,234]. BART transcripts are processed into microRNAs (miRNA) that modulate LMP-1 expression [235] and the ability of infected B cells to avoid immune detection and clearance [236].

Latent viral gene expression is regulated by differential promoter utilization and is controlled by epigenetic marks to both DNA-bound histones as well as the DNA itself [33,237]. In general, loci that are active during latency display unmethylated DNA and acetylated histones, whereas repressed loci display methylated DNA and H3K9 trimethylated histones [238-240]. The molecular mechanisms that lead to these epigenetic marks have not been described. The binding of the chromatin insulator CTCF protein has also recently been shown to modulate viral gene expression during latency [241,242]. As mentioned earlier, lytic viral gene expression during latency is suppressed by genome methylation. In addition, expression of the Z protein is specifically inhibited by the cellular transcription factor Zeb1 [243,244].

Reactivation of natural EBV infections (Figure 3C) occurs when infected memory B cells differentiate into plasma cells in response to antigen stimulation [29,214,229]. This can be mimicked *in vitro *by cross-linking of the B cell receptor by treatment with an anti-immunoglobulin antibody [245,246]. The activation of cellular transcription factors BLIMP1 and XBP-1 upon differentiation likely plays a role in facilitating viral lytic phase gene expression [247-249]. Reactivation is also commonly initiated *in vitro *by the transfection of an expression plasmid for the Z protein or by treating cells with a combination of the phorbol ester TPA and the HDAC inhibitor sodium butyrate [245,250,251]. Z protein function is absolutely required for reactivation, as a mutant Z protein, Z(S186A), fails to induce reactivation [252,253]. Unlike during the establishment of EBV latency, the viral genome at the time of a reactivation event is methylated, and thus the newly expressed Z is able to efficiently activate the expression of the viral early and late genes and thereby promote the productive, lytic replication program.

### Animating Latent EBV Infections

Z expression is necessary and sufficient for the reactivation of latent EBV infections [214,254,255]. Artificial downregulation of the cellular Zeb proteins that silence Z during latency induces Z expression and reactivation [243,244]. Furthermore, microarray data mining indicates that Zeb mRNA levels decrease quickly and precipitously after antigen-mediated differentiation of B cells into plasma cells (Janet Mertz, personal communication). Thus, it is distinctly possible that induction of Z expression is the animating event during EBV reactivation, and that this may in part occur by the disappearance of cellular repressor proteins that silence Z expression during latency. However, recent evidence indicates that *de novo *gene expression is required in order to induce Z expression upon B cell differentiation [256]. Production of Z transcripts following cross-linking of the B cell receptor was prevented by protein synthesis inhibitors, leading authors of that study speculated that a newly synthesized cellular protein was responsible for turning on Z expression. However, in analogy to HSV-1 (and perhaps HCMV) animation, it is distinctly possible that the *de novo *expression of a tegument protein, perhaps BNRF1, is the actual event prevented by the protein synthesis inhibitors that impair EBV reactivation. It would be interesting to see if the BNRF1-null virus fails to reactivate from latently infected B cells upon receptor crosslinking, and if the Zeb proteins also modulate BNRF1 expression. If this were true, animation would likely be the only step during reactivation that requires BNRF1, because the null virus was proficient for reactivation from 293 cells upon ectopic Z expression [220].

## Conclusions

Establishment of latency (Figure 1) for HSV-1 and HCMV appears to be quite similar. Viral IE genes are not expressed because the tegument transactivators required for that event are restricted from entering the nucleus. Other viral gene expression may not be required for the establishment of latency. The major difference is that the silencing of IE gene expression for HSV-1 appears to result from a lack of promoter activation, whereas for HCMV it results from both active promoter repression by cellular factors such as Daxx and HDACs as well as by an unidentified trans-dominant, HDAC-independent mechanism [153]. The establishment of EBV latency is significantly different. It requires viral gene expression, including lytic phase genes of the IE and early classes, to promote cellular proliferation and prevent apoptosis [202-204]. It is presently unclear if or how viral tegument proteins activate this gene expression.

Maintenance of latency (Figure 2) is significantly different for each virus, although assembling a repressive chromatin architecture on the promoters of lytic phase genes appears to be a common control mechanism [20-22,27,33,257]. HSV-1 remains latent in a non-dividing cell and thus does not need to replicate or faithfully partition its genome. Significant control measures during latency appear to be miRNA mediated silencing of any spurious IE gene expression that may occur [103,108], and NGF-mediated Akt phosphorylation that inhibits reactivation by an unknown mechanism [34]. Inhibition of apoptosis may also be important, as is the quelling of reactivation events in a non-cytolytic manner by interferon gamma and granzyme B mediated degradation of ICP4 [258]. HCMV appears to express at least some proteins during the maintenance of latency. Thus, not surprisingly, at least one (vIL10) appears to limit immune detection and clearance of latently infected cells [186,187]. Roles for other viral proteins expressed during latency are not known. Likewise, it is unclear if latently infected cells divide, and thus it is also unknown if mechanisms for genome replication or partitioning exist or are required. miRNA mediated silencing of IE gene expression during latency has been proposed but not demonstrated. EBV expresses multiple genes during latency, many of which ensure cell survival and proliferation. In addition, EBNA1 promotes replication of the viral genome and equal partitioning to daughter cells during cell division. Most lytic phase genes are kept silent by DNA methylation at elements within their promoter regions.

Animation of latency (Figure 3) has been most extensively characterized for HSV-1, where *de novo *expression of the tegument transactivator VP16 appears to be the initiating step of the reactivation process [130]. EBV also requires *de novo *protein expression prior to synthesis of its IE gene encoding the Z protein during latency animation [256], but whether the required protein(s) is viral or cellular (or both) is not known. Thus, whether or not EBV and/or HCMV genomes are animated by *de novo *expression of tegument transactivator remains to be determined. Interestingly, recent experiments indicate that the HCMV tegument transactivator pp71 is a target of granzyme M mediated cleavage [259]. Thus it is possible that if *de novo *expression of pp71 is an animating event for HCMV, granzyme mediated protein cleavage may help extinguish HCMV reactivation events as it appears to do for HSV-1. Reactivation for all herpesviruses likely begins at or prior to IE gene expression, but then continues with a similar kinetic cascade of gene expression that is observed during *de novo *lytic infections. Interestingly, HSV-1, HCMV, and EBV each encode IE proteins (ICP0, IE1, and Z, respectively) that disrupt PML-NBs, nuclear structures that suppress the lytic replication of DNA tumor viruses [260-263], indicating that proteins that localize to these structures may also play significant roles during the establishment and/or maintenance of latency [147]. Thus, the molecular mechanisms of animation and reactivation for the individual herpesviruses, although initiated by different stimuli, may be more conserved than currently appreciated.

## Competing interests

The authors declare that they have no competing interests.

## Authors' contributions

RRP and RFK wrote the manuscript. All authors have read and approved the final manuscript.
